# Application of Gelatin Decorated with Allura Red as Resonance Rayleigh Scattering Sensor to Detect Chito-Oligosaccharides

**DOI:** 10.3390/md18030146

**Published:** 2020-02-29

**Authors:** Weiling Zou, Zijun Sun, Zhengquan Su, Yan Bai

**Affiliations:** 1 School of Public Health, Guangdong Pharmaceutical University, Guangzhou 510310, China; weilingzouzs@163.com (W.Z.); sunzijunlm@163.com (Z.S.); 2 Guangdong Engineering Research Center of Natural Products and New Drugs, Guangdong Provincial University Engineering Technology Research Center of Natural Products and Drugs, Guangdong Pharmaceutical University, Guangzhou 510006, China

**Keywords:** chito-oligosaccharides, allura red AC, gelatin, triple-wavelength overlapping resonance Rayleigh scattering

## Abstract

A convenient and sensitive triple-wavelength overlapping resonance Rayleigh scattering (TWO-RRS) method for the detection of chito-oligosaccharides (COS) was proposed based on enhancing the rigid surface of porous reticular spatial structure of gelatin and COS by introducing allura red AC (AR). The interaction and resultant porous reticular spatial structure were characterized with transmission electron microscopy (TEM), RRS, and UV-Vis spectroscopy. The results indicated that gelatin and COS formed porous reticular spatial structure with an average diameter of 1.5–2.0 μm, and the RRS value of COS-AR-gelatin ternary system with gelatin participation was significantly higher than that of COS-AR binary system. Under the optimal conditions, the enhanced TWO-RRS intensity of the system was linearly proportional to COS concentration in the range of 0.30–2.50 μg/mL, and the regression equation was Δ*I* = 4933.2*c* − 446.21 with *R*^2^ = 0.9980. The limit of detection was 0.0478 μg/mL. So, a new method for the detection of COS was established and verified in the health products with satisfactory results.

## 1. Introduction

The degraded products of chitosan or chitin, chito-oligosaccharides (COS) is a kind of low molecular weight oligosaccharide with good water solubility, high bioactivity and easy to be absorbed by human body because of shorter chain lengths and free amino groups in D-glucosamine units [1]. COS captured much attention from researchers on account of its good dissolvability [2] and a higher degree of deacetylation [3]. To our knowledge, COS has been proven to not only have antioxidant activity [4], and antimicrobial activity [5] but also have anti-tumor activity [6,7] and anti-inflammatory properties [8,9]. Therefore, COS was widely used in many fields, such as cosmeceutical products [10], nutraceuticals [11], pharmaceuticals [3], and core-shell nanoparticles [12]. Hence, the establishment of an accurate analysis method of COS has important practical significance for the quality control of COS products.

The present methods for the detection of COS include spectrophotometry [13], high performance liquid chromatography (HPLC) [14], ion chromatography [15], enzyme-linked immunosorbent assay (ELISA) [16], and capillary electrophoresis [17]. However, most of these methods have some limitations. Spectrophotometry has the advantages of low cost and simple operation, but always needs to hydrolyze COS into monomers before colorimetric detection, so the accuracy of the results will be affected by incomplete hydrolysis. The ELISA has the advantages of rapidity, and high reproducibility, but the sensitivity and accuracy is largely dependent on the antibodies used. Capillary electrophoresis, ion chromatography, and HPLC methods can accurately quantify samples by using COS with different degrees of polymerization (DP) as standard, but these methods are mainly suitable for detecting COS with the DP of 2 to 6 (Table 1), and obtaining the COS standard of a single DP is the first obstacle to be overcome. Therefore, the development of a simple, rapid, sensitive and accurate quantitative analysis method will greatly improve the quality control level of COS products.

It is noteworthy that resonance Rayleigh scattering (RRS) has been widely applied to quantitative analysis [18,19,20,21,22] due to its sensitivity, rapidity and simplicity. In recent years, single-wavelength resonance Rayleigh scattering (SW-RRS) methods [23,24,25,26] were widely used in research, but more scholars were gradually adopting multi-wavelength resonance Rayleigh scattering (Multi-RRS) research. For example, Ma, C. et al. proposed for the detection of chitosan in health products with Brilliant Blue with the double-wavelength overlapping resonance Rayleigh scattering (DWO-RRS) [27] and a DWO-RRS method has been reported based on the reaction of the three β-adrenergic blockade drugs with erythrosine B (EB) to form ion-association composites, which resulted in the RRS intensity enhanced significantly with new scattering peaks appearing at 337 nm and 370 nm, respectively [28]. Xiu Li Hao et al. reported a triple-wavelength overlapping resonance Rayleigh scattering (TWO-RRS) method for the detection of dextran sulfate sodium [29]. Using ethyl violet (EV) as probe, a sensitive and selective method based on the TWO-RRS method for the detection of trace AR in beverage samples was established [30]. Multi-RRS methods have higher sensitivity than SW-RRS methods. However, up to now, there have been few reports on the detection of COS by Multi-RRS method.

Gelatin is a denatured fibrin formed by partial thermal hydrolysis of collagen, with a molecular weight of tens of thousands. Due to its unique functions and process properties, it was widely used in food, medicine, cosmetics, and photography [31].

The interaction between chitosan or COS and gelatin formed a porous reticular spatial material with loose structure, but the material has good adsorption properties. Therefore, it was widely used as a carrier for adsorbing pollutants [32,33,34,35]. However, the application of structural properties of COS-gelatin in analytical chemistry has not been reported. The present study found that the porous reticular spatial structure formed by COS and gelatin was relatively loose, and the RRS value of the solution system was relatively low. On this basis, the allura red AC (AR) with rigid planar structure was introduced in this study. The sulfonic groups of AR molecules associated with the protonated amino group in COS and gelatin molecules formed a multi-molecule reticular composite with rigid planar structure, which improved the strength of RRS greatly.

AR is an azo dye with a benzene ring and a naphthalene ring which has high planar and was used as a probe for the detection and analysis. So far, AR has not been used as a probe for applying in RRS or quantitative analysis of COS.

In this paper, the interaction among COS, gelatin and AR was studied. At the same time, RRS properties of the COS-AR (mixed system of COS and AR) were compared in the presence or absence of gelatin. It was found that the existence of gelatin increased the RRS intensity of COS-AR system, and within a certain range of COS concentration, the change of RRS value at 344 nm had a good linear relationship with COS concentration. Based on this, a simple, rapid and sensitive method for quantitative analysis of COS was established. The present method has been applied to the quantitative analysis of COS in various health products with satisfactory results.

## 2. Results and Discussion

### 2.1. RRS Characteristics

**Figure 1:**
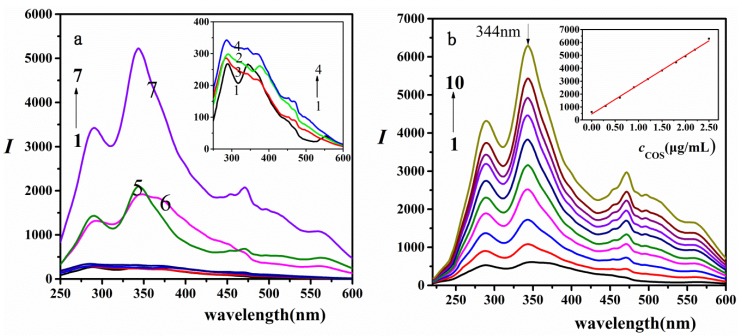
Spectra of COS-gelatin-AR system (**a**) (1) BR-AR; (2) BR-COS; (3) BR-gelatin; (4) BR-COS-gelatin; (5) BR-gelatin-AR; (6) BR-COS-AR; (7) BR-COS-gelatin-AR. 0.50 mL pH = 3.50 BR buffer solution, COS (2.00 μg/mL), AR (1.50 × 10^−5^ mol/L), gelatin (1.50 × 10^−2^ g/L); (**b**) resonance Rayleigh scattering (RRS) spectra of different concentrations of COS. 0.50 mL pH = 3.50 BR buffer solution, COS (from 1 to 10): 0, 0.30, 0.60, 0.90, 1.20, 1.50, 1.80, 2.00, 2.2, 2.5 μg/mL; AR (1.50 × 10^−5^ mol/L); gelatin (1.50 × 10^−2^ g/L).

As shown in Figure 1a, the RRS intensities of COS, gelatin, AR, COS-gelatin (mixed system of COS and gelatin), and gelatin-AR (mixed system of gelatin and AR) in BR buffer solution with pH 3.50 were very weak under the experimental conditions. When COS and gelatin form binary composite COS-gelatin, the RRS intensity was not enhanced for the reason that the COS-gelatin composite was a three-dimensional reticular spatial structure with many pores reticular spatial. When AR was introduced into the system, the ternary composite of COS-gelatin-AR(mixed system of COS, gelatin and AR) was formed, which led to the significant increase of RRS values and the response of the RRS value to COS concentration in COS-gelatin-AR ternary system was much greater than that in COS-AR(mixed system of COS and AR) binary system. The largest scattering peak of the RRS was at 344 nm, followed by 289 nm. Under appropriate conditions, the content of COS was proportional to the enhancement of RRS (Figure 1b) and when the concentration of AR was 1.50 × 10^−5^ mol/L and gelatin was 1.50 × 10^−2^ g/L, there was a good linear relationship between the RRS intensity and the concentration of COS. Therefore, based on the linear enhancement of RRS value with the increase of COS concentration, a method for detection COS was established.

### 2.2. Optimization of the Experimental Conditions

#### 2.2.1. Effects of Acidity

**Figure 2:**
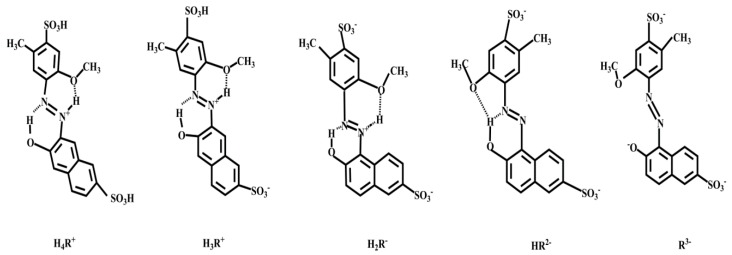
Plane structure diagram of allura red AC (AR) under acidic conditions.

For the sulfonate anion of AR to form an ionic association with the amino cation of COS, the system required a buffer solution to provide a suitable acidity environment. To our knowledge, there are two sulfonic groups in the AR molecule structure and the sulfonic group has strong acidity, so it is easy to dissociate hydrogen ions and make the group negative charged. The protonation degree of amino cations in the COS molecule increases with the decrease of pH value. On the contrary, under alkaline conditions, the structure of AR is R^3−^ with dihedral (Figure 2) due to the electrostatic repulsion of negative charge fragments of methoxy and naphthol anions. The formation of this structure is also related to the slight rotation of the benzene ring [36]. This molecular structure was not conducive to enhancing the RRS signal. What is more, with the increase of pH, the degree of amino protonation of COS decreased gradually, and the ion association between COS and AR decreased gradually. 

In this study, the effects of four buffer solutions of citric acid-sodium citrate, disodium hydrogen phosphate-citric acid, BR buffer solution and glycine-hydrochloric acid on the RRS of COS-AR system were investigated. The results showed that in BR buffer solution, the linear relationship between the concentration of COS and Δ*I* was fine and the sensitivity was high, so BR buffer solution was chosen as the buffer medium.

The BR buffer solutions with pH 2.50 to 7.00 were selected. The results showed that the RRS value of the system increased with the decrease of pH, and tended to be stable when the pH was less than 4.0 (Figure S1a).

With regard to the reasons, on one hand, as the amino of COS was almost completely protonated at pH < 4 [17], the number of amino cations was no longer increased even though the pH was further reduced. On the other hand, because there were two pairs of lone pairs of electrons in the outermost layer of an oxygen atom in methoxy group, and in acidic condition, the outer orbit of azo protonated hydrogen ion was empty. They would form intramolecular hydrogen bonds, thus increasing the planarity of AR. The Kateryna Bbvziuk research [36] also showed that when pH was less than 4.00, the molecular structures of AR after protonation at different pH values were different, but they were all rigid planar structures (Figure 2). So, when the system was under acidic conditions, the RRS value had a platform.

Therefore, in order to stabilize the detection system in the optimal acidity state, the final choice of pH 3.50 was the follow-up experimental acidity. Because excessive buffer solution would be unfavorable for RRS detection due to salt effect, the adding amount was chosen 0.50 mL (Figure S1b).

#### 2.2.2. Effect of the Concentration of AR

According to Figure S1c, when AR concentration was less than 1.0 × 10^−5^ mol/L, due to the insufficient amount of AR, it could not fully interact with the amino cations of COS, so the RRS value of the solution system was relatively low. With the increase of AR concentration, the ion association of amino cations in COS tended to be complete, and the RRS value of the solution system tended to be stable.

Figure 1a also showed that the RRS value in the presence of AR alone in solution was very weak, and there was no obvious change with the increase of AR concentration. Therefore, the subsequent experiments were carried out with the AR concentration of 2.00 × 10^−5^ mol/L.

#### 2.2.3. Effects of Gelatin

When gelatin was heated above the critical temperature of gelation, the intramolecular hydrogen bonds of gelatin were destroyed, so gelatin dissolved in water and presented a random crimp structure to form large cavities. At this time, the polar groups of gelatin formed a porous reticular spatial structure with COS via hydrogen bonds [37,38]. Because of the loose reticular spatial structure and weak interfacial property of molecular beams, its RRS values were relatively low. However, under the proper concentration, it provided a large planar basis for further ion association with AR. By combining the planar structure of AR with the porous reticular spatial structure of COS-gelatin, a more compact planar structure was formed, which significantly enhanced the RRS values of the system.

In order to further explore the effect of gelatin on the detection system, the differences between gelatin and gelatin-free in the COS-AR system were compared. It was found that the presence of appropriate gelatin could significantly enhance the strength of RRS in COS-AR system (Figure S1d). When the concentration of gelatin was 1.50 × 10^−2^ g/L, the RRS value of the system was the strongest.

With the increase of gelatin content, due to the adsorption between gelatin and COS, the main molecules exposed to the outer layer of COS-gelatin molecular micelle were gelatins. Because the ion association between COS-gelatin molecular micelles and AR was mainly caused by sulfonic anions of AR and amino cations of COS, it was more difficult to associate COS with AR ions due to the existence of steric hindrance when the molecular beam interacted with AR. Therefore, with the increase of gelatin content, the RRS of COS-gelatin-AR (*I*) decreased. At the same time, with the increase of gelatin concentration, the porous plane structure of gelatin-AR increased, so the RRS value of gelatin-AR (*I*_0_) increased gradually, and then Δ*I* value decreased greatly.

#### 2.2.4. Effects of Temperature and Interaction Time

The heating temperature was selected as room temperature (30 °C), 40 °C, 50 °C, 60 °C, 70 °C, 80 °C, 90 °C, and 100 °C eight gradients and heating time was 10 min. The results showed that when the temperature was 60 °C, the system reached the maximum RRS value, so the optimal interaction temperature of the system was 60 °C (Figure S2).

At a certain temperature, COS and gelatin formed a porous spatial reticular structure through hydrophobic interaction and intermolecular hydrogen bonding in solution, and then associated with AR ions to synthesize dense and coplanar molecular beams. Because increasing the temperature properly was helpful to the thermal movement of the molecule, the formation of COS-gelatin-AR ternary composite was promoted.

However, according to Gibb’s free energy formula ΔG=H−TΔS, the formation of COS-gelatin-AR ternary composite would result in the decrease of solution entropy, and that was ΔS < 0. Considering that the possibility of chemical reaction between various substances was small, the enthalpy change of physical adsorption between COS and gelatin was uncertain, ΔH usually was less than zero. Combined with the formula ΔG=H−TΔS, it could be concluded that high temperature will be unfavorable to the adsorption of COS and gelatin. So, at lower temperature, the adsorption could proceed spontaneously, but with the increase of temperature, the value of ΔG gradually approached zero, and the spontaneity of inter-molecular adsorption gradually weakened. When ΔG was greater than zero, the ternary composite of COS-gelatin-AR would not be formed. Therefore, it could be seen from Figure S2 that the RRS intensity of the solution system gradually increased, and reaching the maximum at 60 °C, and the RRS values of the system decreased significantly under high temperature. The effect of interaction time on the RRS of the system was further investigated. The results showed that the Δ*I* of the system decreased slightly with the increase of interaction time, so the subsequent experiment selected 60 °C water bath for 5 min.

#### 2.2.5. Effects of Sequence and Standing Time

Effect of the adding sequence of different reagents was investigated. The results indicated that the sequence of “BR-COS-gelatin-AR” was the best. Under the optimal condition, the effect of standing time on the stability of RRS intensity was studied. The results showed that the Δ*I* reached the maximum at 5 min after all reagents were added, and it remained stable for over 180 min. Therefore, this system exhibited good stability (Figure S3).

### 2.3. Orthogonal Experimental Design Optimization

On the basis of experimental research, three control factors (independent factors) affecting *I*_RRS_ were considered. The concentration of AR (A), the concentration of gelatin (B), the volume of BR (C) were set up as the controlling factors and the blank column (D) was set up as experiment error at the same time. For each controlling factor, three different levels were selected. The range of choices between the three levels depended on previous laboratory studies, 1.00×10^−5^ mol/L, 1.50 × 10^−5^ mol/L and 2.00 × 10^−5^ mol/L for A, 1.00 × 10^−2^ g/L, 1.50 × 10^−2^ g/L and 2.00 × 10^−2^ g/L for B, 0.30 mL, 0.50 mL, and 0.70 mL for C. The test conditions were designed according to the L_9_ (3^4^) orthogonal test, and the test index was performed with RRS intensity [19]. The original data was analyzed using an orthogonal experimental design.

The order of the control factors affecting the RRS method and the analysis of variance (ANOVA) was shown in Table 2. The order of influence of the control factor was: A > B > D > C. Then, ANOVA results showed that all controlling factors were insignificant with *p* > 0.05 using SPSS 22.0, which indicated that the changes of the three main influencing factors within a certain range had little impact on the results (Table 3). Thus the optimization of the analytical conditions in our research was 1.5 × 10^−5^ mol/L of AR, 1.5 × 10^−2^ g/L of gelatin and 0.50 mL of BR buffer solution, which was applied in further research.

### 2.4. Effects of Ionic Strength

The effect of ionic strength on the intensities of RRS of the COS-gelatin-AR system was investigated with 0~0.0300 mol/L NaCl and the results showed that the RRS intensities decreased obviously with an increase in NaCl concentration due to the encumbrance of ion–association interaction resulting from the high concentration of Cl^−^ and Na^+^ (Figure S4). The results also indicated that the electrostatic interaction was a very important factor in this ion–association interaction. The association interaction should be under a low ionic strength condition [39]. Therefore, the whole experimental water was deionized water, and the sample pretreatment used dialysis to remove excess ion interference.

## 3. Mechanism for Molecular Interaction and Reasons for RRS Enhancement

### 3.1. Mechanism

Firstly, because of the p*K*a of the gelatin was 4.50–4.75, the molecules of gelatin were relatively curly, while in the acidic and alkaline media on both sides of the p*K*a, the molecules of gelatin were relatively stretched for electrostatic repulsion [40]. Secondly, when gelatin was heated at high temperature, intramolecular hydrogen bonds and hydrophobic forces between macromolecules were destroyed. The residual amino acids were exposed and interacted with other groups, forming hydrogen bond with water molecules to accelerate dissolution [41]. Therefore, the influence of heating and acidic environment made gelatin tape positive and dispersed in solution.

When pH was 3.50, the amino groups in COS were protonated, and the carboxyl groups of glutamic acid and aspartic acid residues of gelatin were slightly dissociated into anions, amino cations, and carboxyl anion groups would interact through electrostatic attraction [42]. In addition, because both gelatin and COS have long hydrophobic chains, the hydrophobic interaction force would promote the aggregation of the two macromolecules, thus cross-linking the long-chain COS with the long-chain gelatin and forming a positive charged porous reticular spatial structure.

Subsequently, when the pH of research system was 3.50, the structure of the AR was shown HR^2−^ in Figure 2. Since the gelatin and COS formed a three-dimensional porous reticular spatial structure, and AR had a rigid plane, which was combined with the porous reticular spatial structure by electrostatic attraction to form a larger and compact rigid plane, so the RRS increased sharply.

**Scheme 1:**
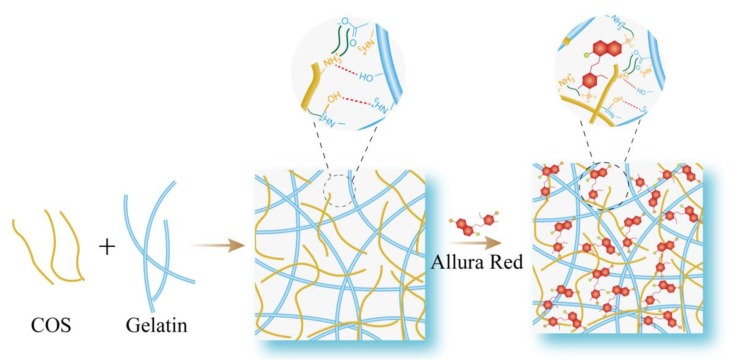
Schematic illustration the mechanism scheme of COS, gelatin, and allura red.

Therefore, according to the above experimental and analytical results, in this study, we designed a new optical sensor based on RRS enhancement. By introducing AR to enhance the rigid plane of COS-gelatin with porous reticular spatial structure, the RRS value of the system was significantly increased, and the RRS response value was linearly related to the concentration of COS, so as to detect COS. The proposed novel strategy for COS detection was shown in Scheme 1. And the carboxyl group and hydroxyl group in gelatin could easily form hydrogen bonds with water, which also increased the stability of the molecular beam.

### 3.2. The Reasons for RRS Enhancement

#### 3.2.1. Increased Rigid Plane

**Figure 3:**
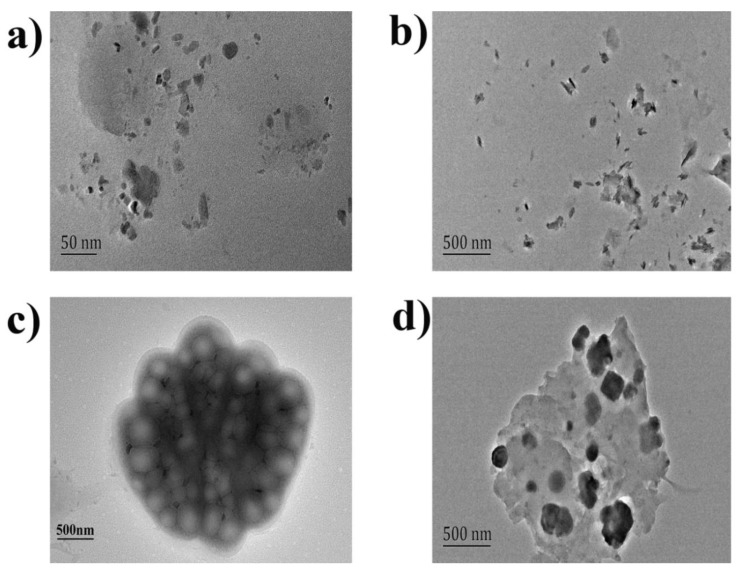
TEM of the AR, COS-AR, COS-gelatin and COS-gelatin-AR. (**a**) 0.50 mL pH = 3.50-BR buffer solution and 1.5 × 10^−5^ mol/L AR; (**b**) 0.50 mL pH = 3.50 BR buffer solution, 2.50 μg/mL COS and 1.5 × 10^−5^ mol/L AR; (**c**) 0.50 mL pH = 3.50 BR buffer solution, 2.50 μg/mL COS and 1.50 × 10^−2^ g/L gelatin; (**d**) 0.50 mL pH = 3.50 BR buffer solution, 2.50 μg/mL COS, 1.50 × 10^−2^ g/L gelatin and 1.5 × 10^−5^ mol/L AR.

The diameter and shape of the formed nanoparticles were observed by TEM. Figure 3a showed that the shape of the AR particles was irregular, with a diameter about 25 nm. After AR was combined with COS (Figure 3b), the diameter of the compound COS-AR particles increased, with a diameter about 200–250 nm. From Figure 3c, gelatin interacted with COS to form the porous reticular spatial structure with a diameter about 1.5–2.0 μm, which had the cavity with low electron cloud density. Although the volume of the reticular structure formed by gelatin and COS was larger, the RRS was relatively poor due to the loose cavity structure with the interfacial property. However, when AR was interacted with COS-gelatin system by electrostatic attraction, the dense ternary composite was formed, and many co-planars were added on the basis of porous spatial reticular structure (Figure 3d). The above results showed that the RRS values of the system increased with the increase of the diameter and planarity of the molecular beam in the solution.

According to Figure 3 the average diameter of COS-gelatin-AR composite was approximately the same as that of COS-gelatin, about 1.5 µm, but the RRS strength of them was quite different. The results showed that although the composite with gelatin was even in the size of microns, the resonance scattering should occur within the nano-particles in the composite, and the RRS strength of the analytical system was more closely related to the micro molecular structure of the molecular beam.

#### 3.2.2. Scattering–Absorbing–Rescattering

**Figure 4:**
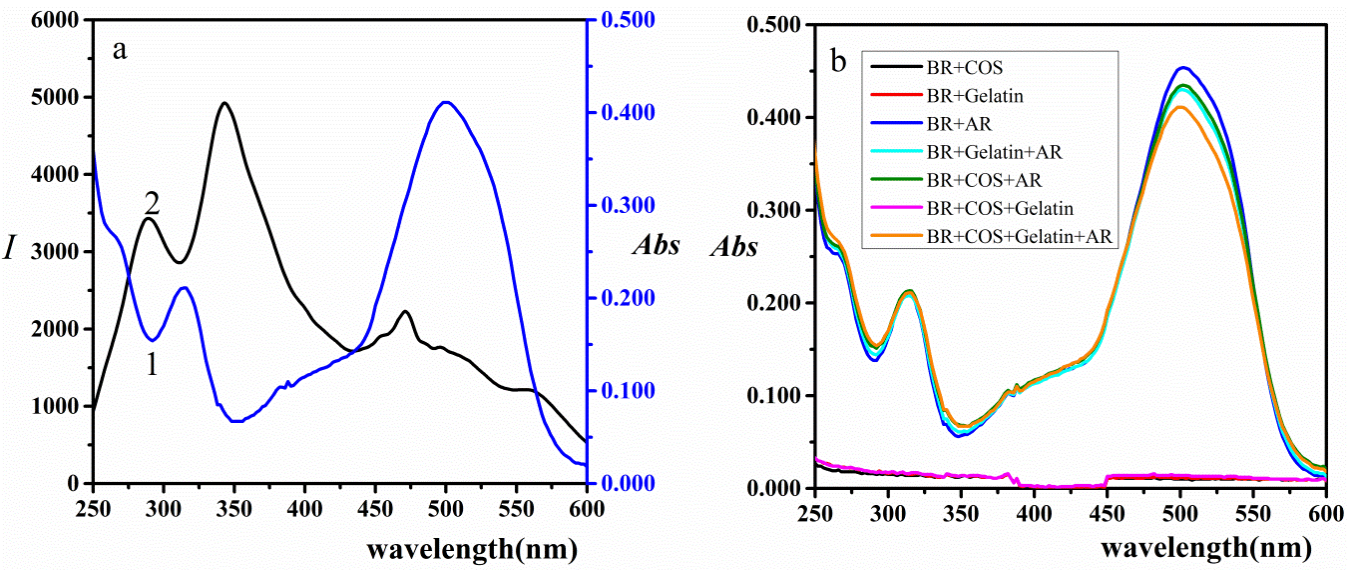
(**a**) The comparison of absorption spectra and RRS spectra of COS-gelatin-AR. 1. Absorption spectra; 2. RRS spectra. 0.50 mL pH = 3.50 BR buffer solution, COS (2.00 μg/mL), AR (1.50 × 10^−5^ mol/L), gelatin (1.50 × 10^−2^ g/L). (**b**)Absorption spectra of COS-gelatin-AR system. 0.50 mL pH = 3.50 BR buffer solution, COS (2.00 μg/mL), AR (1.50 × 10^−5^ mol/L), gelatin (1.50 × 10^−2^ g/L).

When the RRS band of COS-gelatin-AR system was located at or close to its absorption band, electrons absorbed electromagnetic wave at the same frequency as scattering frequency. Because of the resonance of the scattering light and the absorption light at the same frequency, electrons strongly absorbed the energy of light and generate re-scattering, which resulted in the significant enhancement of the RRS intensity [19,43]. As shown in Figure 4a, the three resonance scattering peaks at 289 nm, 344 nm, and 471 nm were all in the molecular absorption band of COS-gelatin-AR system. Figure 4a also showed that the two resonance light scattering peaks at 289 nm and 344 nm corresponded to the bottom of the molecular absorption valley at 288 nm and 350 nm. There was a large molecular absorption peak near the 471 nm scattering peak, where the RRS intensity was also low.

Combining the information of Figure 4a and the scattering cross section’s formula [44,45] as below, the relationship between the molecular absorption and the RRS intensity of the COS-gelatin-AR ternary composite was explored.

(1) $$Q_{i}=\frac{\sigma_{i}}{\pi a^{2}}$$

where *a* is the radius of the particle, *Q* represents extinction, absorption and scattering efficiency (*Q_ext_*, *Q_abs_*, and *Q_sca_*) and *σ* is the cross-sectional area surrounding a particle and *i* is sca, abs, ext, and pr, representing scattering, absorption, extinction, and radiation pressure, respectively. The intensity of the scattered light is proportional to the light cross section of the particle. The combined processes of absorbing and scattering light are referred to as light extinction [46]. According to the law of conservation of energy:

*Q_ext_* = *Q_sca_* + *Q_abs_* or *σ_ext_* = *σ_sca_* + *σ_abs_* (2)

When the intensity of the absorbed light decreased, the intensity of the scattered light increased. Since the scattering and absorption were at the same frequency, part of the absorbed energy was scattered due to resonance, which resulted in enhanced scattered light.

From Figure 4b, the absorption spectra also showed that AR, COS-AR, gelatin-AR, and COS-gelatin-AR all had absorption. When COS or gelatin was added to AR solution, the absorption was slightly decreased. However, when COS bound with gelatin and then interacted with AR, the absorbance of the solution reduced more, which implied that there was a special interaction between COS and gelatin, and then bound with AR to form ternary composite.

#### 3.2.3. Enhancement of Hydrophobicity

Under the experimental conditions, the hydrophilic groups of COS, AR, and gelatin made them dissolve in water to form hydrates, and the scattering intensity of single substance was weak. However, when they interacted to form ternary composite COS-gelatin-AR, the hydrophobicity was enhanced for the electrical neutrality and hydrophobicity of the ionic association, which also enhanced scattering intensity. Due to the presence of hydrophobic long-chain alkyl and aryl skeletons of AR, the ternary ion composite had a large hydrophobic structure and a molecular co-planarity, which results in strong interfacial scattering effect and enhanced scattering intensity.

## 4. Selectively and Analytical Applications

### 4.1. Selectively

Based on the above experimental conditions, the influence of 20 coexisting substances on the COS-gelatin-AR system was investigated and when the relative error of about 5% was calculated, the maximum concentration of the coexisting substance allowed to be added was calculated (Table 4). The results showed that common amino acids, some sugars, cationic K^+^, Zn^2+^, Mg^2+^, etc., had less interference, and the allowable amount was larger while glucosamine and some metal ions Ca^2+^, Hg^2+^, Fe^3+^, and Al^3+^ had a greater influence on the results so the allowable amount was relatively small. By comparing the results, it was found that the substances that interfered greatly with the detection system were all small molecular substances.

Therefore, in the detection of actual samples, this method was combined with the dialysis pre-treatment operation to remove the ionic state or small molecule which greatly interfered with the detection results, so as to obtain more satisfactory results.

### 4.2. Linear Range and Detection Limit

Under optimal experimental conditions, COS interacted with gelatin and AR in solution. The RRS intensity of the solution was measured, and the reagent blank *I*_0_ was also determined. Taking the COS concentration as the abscissa and Δ*I* as the ordinate, the standard curve was drawn and the regression equation was calculated. Table 5 listed the results of TWO-RRS and RRS methods. TWO-RRS method showed a higher sensitivity and lower limit of detection (LOD). The results showed that the linear range of the method was 0.30–2.50 μg/mL, and the regression equation was Δ*I* = 4933.2*c*−446.21 with *R*^2^ = 0.9980 under TWO-RRS, and the detection limit was 0.0478 μg/mL (LOD = 3S_b_/S, S was the slope of the calibration curve and S_b_ was the standard deviation of the corrected blank RRS signals of the reagent blank).

### 4.3. Application

#### 4.3.1. Sample Pretreatment

The sample was accurately weighed 5.00 g in a beaker, dissolved in a small amount of water, transferred to a 100.0 mL volumetric flask, and diluted to the mark with water. The insoluble was then discarded by centrifugation at 6000 r/min for 30 min. Then, 10.00 mL of the supernatant was then dialyzed through a dialysis bag (500MWCO) for 4 h, and then all of the dialysis fluid was transferred into a 100.0 mL volumetric flask and diluted to the mark with high purity water to obtain dialyzate. Then sample working solution with concentration of 50.0 μg/mL was obtained by absorbing 1.00 mL Longma dialyzate and 1.10 mL Keer dialyzate into a 100.0 mL volumetric bottle, respectively, diluted with high purity water to the mark. Before sample pretreatment, COS standard solution was used to calculate the recovery rate of dialysis bags, six copies in parallel. The results showed that the recovery of COS standard dialysis was over 98% after 4 h, and the recovery was satisfactory.

#### 4.3.2. Detection of the Samples

In a series of 10 mL colorimetric tubes, 0.50 mL pH 3.50 BR buffer solution, a series of volume COS standard solution, a certain volume of 5.0 μg/mL gelatin solution and 1.5 × 10^−5^ mol/L AR solution were added in turn, and then the solution was fixed to the scale line with deionized water and shaken evenly. Then, the RRS intensity of each tube was measured, and the corresponding standard curve was regressed with Δ*I* as ordinate and COS concentration as abscissa.

According to the above experimental procedures, two kinds of sample solutions were extracted 1.00 mL and 2.20 mL respectively, and all measurements were carried out in nonduplicate. RRS values were measured and Δ*I* = *I* − *I*_0_ was calculated. Through regression equation calculation, the average content of COS in the two samples was 156.8 mg/g for the Longma tablet, and 59.90 mg/g for the Keer capsule. The relative standard deviation (RSD) of parallel samples was 3.35% and 3.71%, respectively.

Furthermore, the recoveries and repeatability were detected by a standard addition method. The results (Table 6) showed that the method had a good repeatability for the detection of COS and the RSD were 2.59% to 3.00% for the Longma tablet and 1.59% to 2.89% for the Keer capsule. The recoveries of the Longma tablet and Keer capsule were 97.3%~100.1% and 100.3%~100.7%, respectively. Therefore, this method could be applied in the detection of the COS in health products.

## 5. Materials and Procedure

### 5.1. Material and Reagents

A Hitachi F-2500 spectrofluorophotometer (Hitachi Ltd., Tokyo, Japan), equipped with a xenon lamp and a 1.00 mL quartz flow cell, was used for recording scattering spectra at a given wavelength with the slit width of 10 nm and the PMT voltage of 400 V. An UV3010 spectrophotometer (Hitachi Ltd., Tokyo, Japan) was employed in recording the absorption spectra and measuring the absorbance. A PHS-3C pH meter (Shanghai Scientific Instruments Company, China) was used to adjust the pH values of the solution, and a CP124C electronic analytical balance (Ohaus Instrument (Shanghai) Co. Ltd.) was used in this experiment. Transmission electron microscopy (TEM) images were collected with JEM2100F (Japan) with an accelerating voltage 200 kV.

The stock solution of 0.40 g/L COS (Tokyo chemical industry Co. Ltd.) was prepared by mixing suitable amount of COS with water, and the working solution of 10.00 µg/mL COS was prepared by being diluted with water. Allura red AC (CNW technologies) was prepared at a concentration of 1.0 × 10^−4^ mol/L with water. Stock solution of 10.0 g/L gelatin (Sinopharm Chemical Reagent Co., Ltd.) was prepared by weighing 1.00 g of gelatin and being soaked in a small amount of water for 2 h after gelatin was fully expanded and dissolved in a 50 °C water bath. The working solution of 0.10 g/L gelatin was prepared by being diluted with water. COS capsules (Shandong Keer Biomedical Technology Development Co. Ltd.) and COS tablets (Dalian Longma Shengke Trading Co. Ltd.) were purchased from supermarket. The entire experimental water is ultrapure water.

### 5.2. Procedure

In a 10 mL calibrated flask, 0.50 mL pH at 3.50 BR buffer solution, appropriate amount of 10.00 μg/mL COS, 1.50 mL 0.10 g/L gelatin, and 1.50 mL 1.0 × 10^−4^ mol/L AR were sequentially added, then diluted to the mark with water and mixed well. The mixture was heated for 5 min in a 60 °C water bath and cooled naturally to room temperature. The RRS spectra were recorded with synchronous scanning at λ_ex_ = λ_em_ (Δλ = 0 nm) on fluorescence spectrophotometer, measuring the RRS intensity *I*_RRS_ for the ion-association composite and *I*_0_ for the reagent blank at the maximum RRS wavelength (344 nm), Δ*I* = *I* − *I*_0_. All experiments were carried out in triplicate.

## 6. Conclusions

In this study, gelatin under heating conditions was used to form a spatial reticular structure with positive charge with COS under acidic conditions. On this basis, the ternary composite of COS-gelatin-AR with rigid plane was formed by ion association with AR anionic dye, which led to a sharp increase in RRS value of the system. The results showed that the TWO-RRS and RRS intensity of the COS-gelatin-AR system was proportional to the COS concentration, and the system had higher sensitivity and better stability than the COS-AR binary system without gelatin. Therefore, a quantitative analysis method for COS was established. The method is simple, sensitive, accurate and rapid, and suitable for the quantitative detection of COS in various kinds of medical and health products.

## 7. Highlights

1. A new resonance Rayleigh scattering method was established to quantitatively detect chito-oligosaccharides by using gelatin decorated with allura red AC as sensor.

2. The mechanism of interaction between chito-oligosaccharides, allura red and gelatin to increase resonance Rayleigh scattering signal was discussed.

## Figures and Tables

**Table 1:** Comparison of the reported methods for chito-oligosaccharides (COS).

Method	Compound	Linear Range	Limit of Detection (LOD)	References
Spectrophotometry		0–60 μg/mL		[13]
HPLC		9.13–91.3 μg/mL		[14]
ion chromatography	(GlcN)1 ^a^	0.2–11.5 mg/L	3 mg/mL	[15]
	(GlcN)2	0.2–9.8 mg/L	8 mg/mL	
	(GlcN)3	0.2–10.1 mg/L	8 mg/mL	
	(GlcN)4	0.2–10.6 mg/L	11 mg/mL	
	(GlcN)5	0.2–10.6 mg/L	13 mg/mL	
	(GlcN)6	0.2–9.9 mg/L	16 mg/mL	
ELISA				[16]
capillary electrophoresis	(GlcN)2		2 μmol/L	[17]
	(GlcN)3		2 μmol/L	
	(GlcN)4		2 μmol/L	
	(GlcN)5		3 μmol/L	
	(GlcN)6		5 μmol/L	

^a^ representing the product of hydrolysis of COS, glucosamine.

**Table 2:** The analysis of range results.

Controlling Factor	A	B	C	D
Lever 1	269	2583	2395	1139
Lever 2	2228	2303	1897	1985
Lever 3	3658	1270	1863	3032
difference	3389	1313	532	1893
Order of difference	1	3	4	2

**Table 3:** The analysis of the ANOVA results.

Factor	DF	SS	MS	*F	*p*
Total	8	26,163,949			
A	2	17,367,881	8,683,941	3.219	>0.05
B	2	2,867,371	1,433,685	0.531	>0.05
C	2	533,159	266,579	0.099	>0.05
Residual error	2	5,395,539	2,697,769		

*F_0.05 (2, 2)_ = 19.

**Table 4:** The effects of coexisting substance for the method.

Coexistent	Tolerance (mol/L)	Relative (%)	Coexistent	Tolerance (mol/L)	Relative (%)
Oxalic acid	1.98 × 10^−4^	−4.5	Starch	40.0 µg/mL	4.28
Citric acid	4.86 × 10^−4^	−4.66	Thiourea	4.00 × 10^−3^	−0.34
EDTA	2.00 × 10^−4^	−5.61	MgSO4	1.00 × 10^−4^	−2.5
Ascorbic acid	4.54 × 10^−3^	3.31	NH_4_Cl	3.71 × 10^−5^	3.11
Glycine	5.33 × 10^−3^	3.06	ZnSO4	3.48 × 10^−5^	−3.63
L-lysine	6.84 × 10^−4^	2.03	FeCl_3_	1.79 × 10^−5^	−4.3
L-leucine	1.91 × 10^−3^	−3.32	AlCl_3_	3.71 × 10^−5^	3.11
L-aspartic acid	5.26 × 10^−4^	−4.78	CaCl_2_	2.50 × 10^−5^	4.24
Glucosamine	3.60 µg/mL	4.24	HgNO_3_	1.50 × 10^−6^	−3.18
Glucose	5.55 × 10^−4^	1.11	KOH	5.12 × 10^−5^	−4.37

**Table 5:** Analytical parameters of RRS and triple-wavelength overlapping resonance Rayleigh scattering (TWO-RRS) methods.

Method	λ (nm)	Regression Equation (μg/mL)	Linear Range (μg/mL)	*R* ^2^	LOD (μg/mL)
RRS	288	Δ*I* = 1505.4*c* − 43.967	0.30–2.50	0.9985	0.087
RRS	344	Δ*I* = 2318.1*c* − 245.39	0.30–2.50	0.9985	0.089
RRS	471	Δ*I* = 1109.6*c* − 148.19	0.30–2.50	0.9935	0.095
TWO-RRS	288 + 344 + 471	Δ*I* = 4933.2*c* − 446.21	0.30–2.50	0.9980	0.0478

**Table 6:** Quantification of COS in samples.

Simple	Found (μg/mL)	Added (μg/mL)	Total Found (μg/mL)	RSD (%, *n* = 9)	Recovery (%, *n* = 9)
Keer	0.65	0.80	1.45	2.89	100.3
		1.10	1.75	1.59	100.7
Longma	0.78	0.80	1.56	2.59	97.3
		1.00	1.78	3.00	100.1

## Supplementary Materials

The following are available online at https://www.mdpi.com/1660-3397/18/3/146/s1, Figure S1. The effect of acidity, the concentration of AR and the reaction temperature. (a)The effect of pH of BR buffer solution.1.00 mL BR buffer solution, COS (2.00 μg/mL), AR (1.00 × 10^−5^ mol/L); (b) The Effect of amount of BR buffer solution. pH = 3.50 BR buffer solution, COS (2.00 μg/mL), AR (1.00 × 10^−5^ mol/L). (c) The effect of AR concentration. 0.50 mL pH = 3.50 BR buffer solution, COS (2.00 μg/mL); (d) The effect of gelatin. 0.50 mL pH = 3.50 BR buffer solution, COS (2.00 μg/mL), AR (2.00 × 10^−5^ mol/L); Figure S2. The effect of temperature on RRS intensity of COS-Gelatin-AR system. 0.50 mL pH = 3.50 BR buffer solution, COS (2.00 μg/mL), AR (2.00 × 10^−5^ mol/L) and gelatin (1.50 × 10^−2^ g/L); Figure S3. The effect of standing time. 0.50 mL pH = 3.50 BR buffer solution, COS (2.00 μg/mL), AR (2.00 × 10^−5^ mol/L) and gelatin (1.50 × 10^−2^ g/L); Figure S4. The effect of the concentration of NaCl. 0.50 mL pH = 3.50 BR buffer solution, COS (2.00 μg/mL), AR (1.50 × 10^−5^ mol/L) and gelatin (1.50 × 10^−2^ g/L).
