# Supplementary material for: Application of Gelatin Decorated with Allura Red as Resonance Rayleigh Scattering Sensor to Detect Chito-Oligosaccharides
Source: Mar Drugs. 2020 Feb 29;18(3):146. doi: 10.3390/md18030146 (PMC7143526; doi:10.3390/md18030146)
Supplement: Supplementary file 1 [file marinedrugs-18-00146-s001.pdf]

Application of gelatin decorated with allura red as resonance Rayleigh scattering sensor to determine chito-oligosaccharides

Weiling Zou[a], Zijun Sun[a], Zhengquan Su[b,*], Yan Bai[a] and [*]

Note: The resonance Rayleigh scattering spectra were recorded with synchronous scanning at $\lambda_{ex}=\lambda_{em}$ ($\Delta\lambda$=0 nm) on fluorescence spectrophotometer, measuring the RRS intensity I for the ion-association complex and I_0 for the reagent blank at the maximum RRS wavelength (344 nm), $\Delta I=I-I_0$.

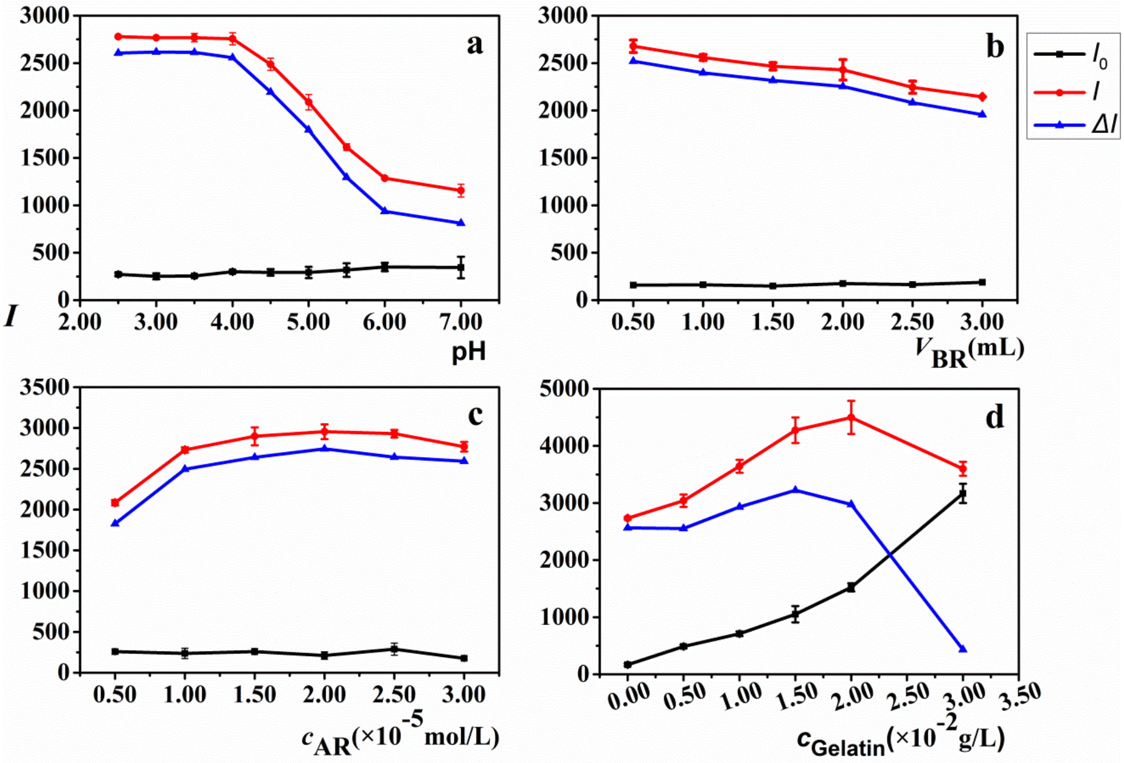

Fig.1S. The effect of acidity, the concentration of AR and the reaction temperature. (a)The effect of pH of BR buffer solution.1.00 mL BR buffer solution, COS (2.00 μg/mL), AR (1.00×10^{-5} mol/L); (b) The Effect of amount of BR buffer solution. pH=3.50 BR buffer solution, COS (2.00 μg/mL), AR (1.00×10^{-5} mol/L). (c) The effect of AR concentration. 0.50 mL pH=3.50 BR buffer solution, COS (2.00 μg/mL); (d) The effect of gelatin. 0.50mL pH=3.50 BR buffer solution, COS (2.00μg/mL), AR (2.00×10^{-5}mol/L)

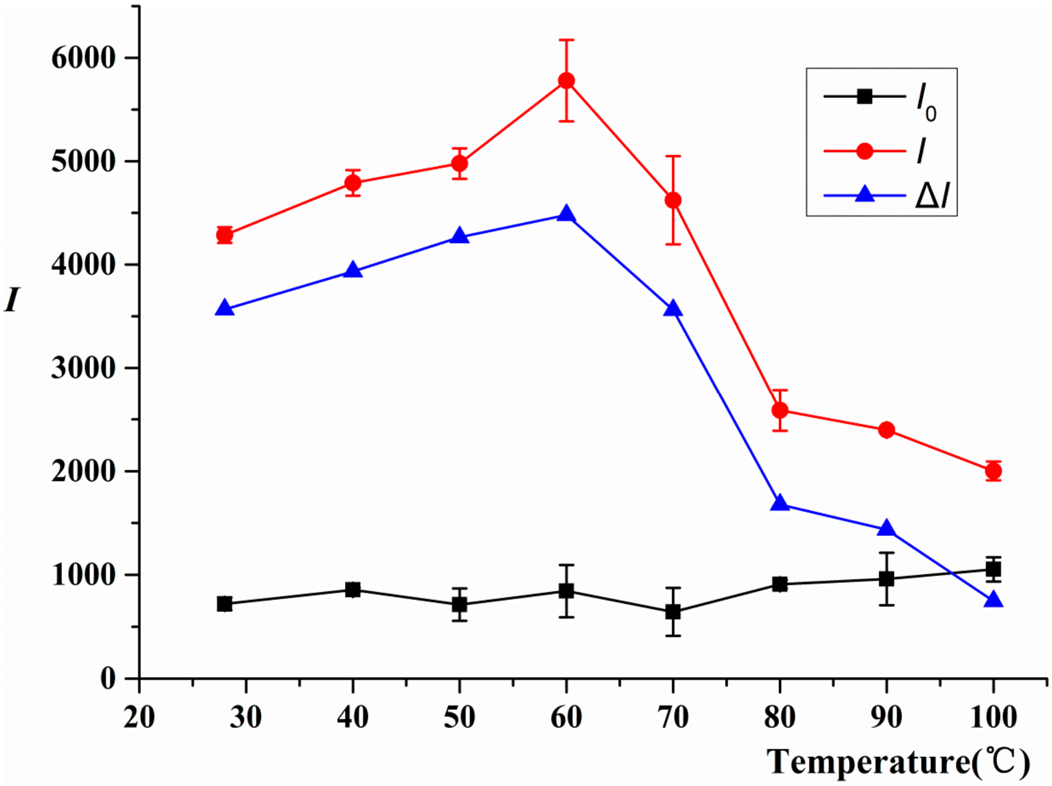

Fig.2S The effect of temperature on RRS intensity of COS-Gelatin-AR system. 0.50 mL pH=3.50 BR buffer solution, COS (2.00 μg/mL), AR (2.00×10^{-5} mol/L) and gelatin (1.50×10^{-2} g/L)

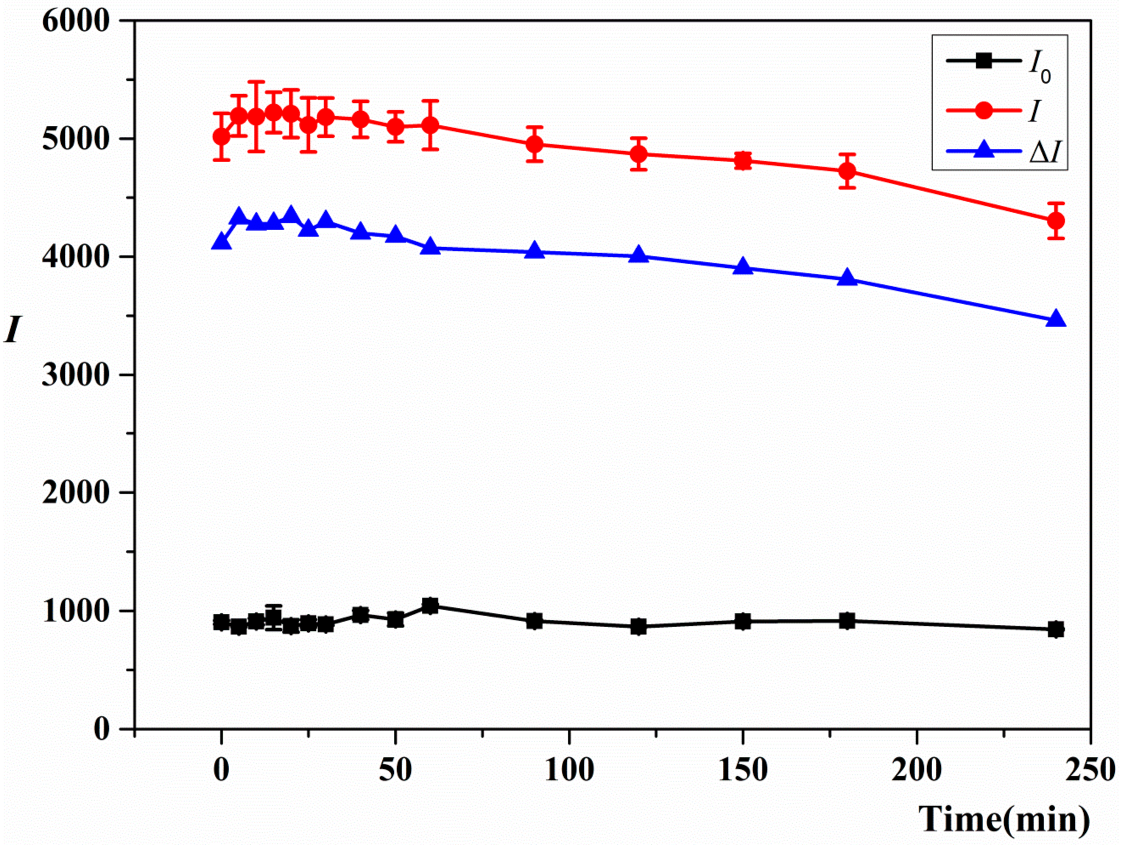

Fig.3S The effect of standing time. 0.50 mL pH=3.50 BR buffer solution, COS (2.00μg/mL), AR (2.00×10^{-5} mol/L) and gelatin(1.50×10^{-2} g/L)

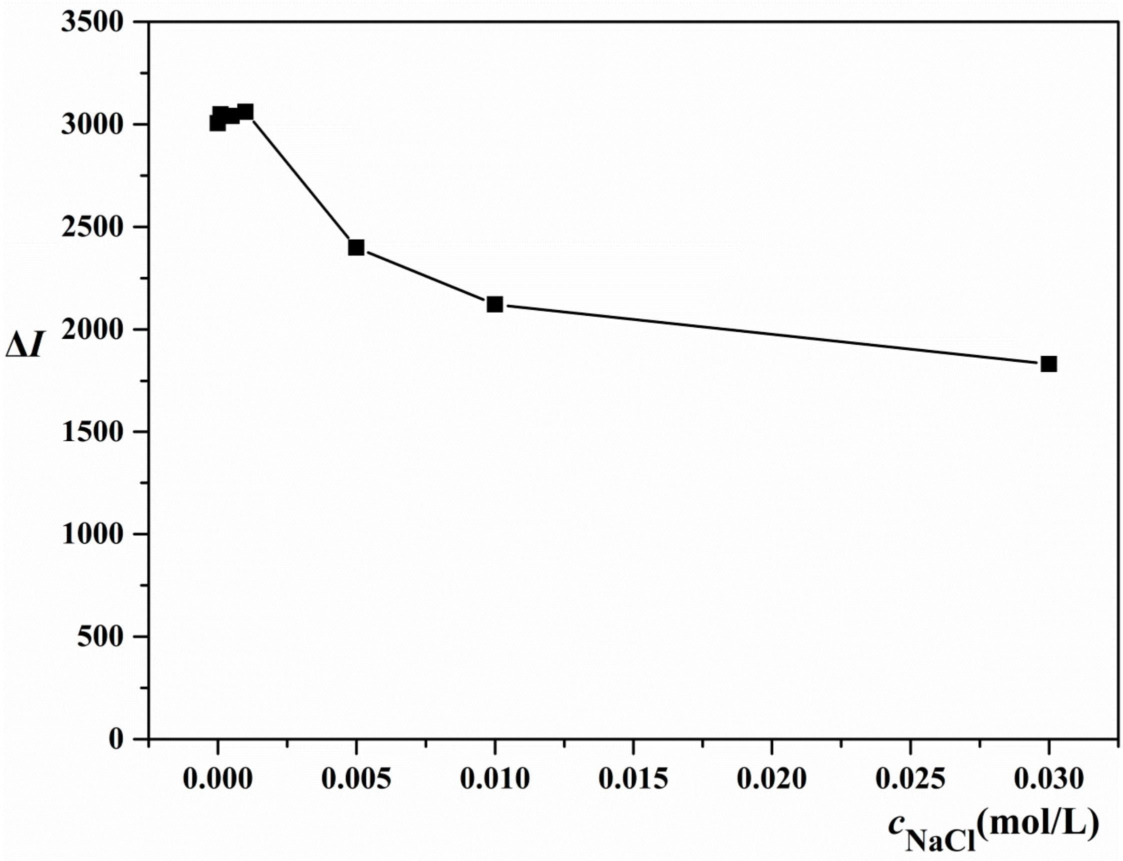

Fig.4S The effect of the concentration of NaCl. 0.50 mL pH=3.50 BR buffer solution, COS (2.00 μg/mL), AR (1.50×10^{-5} mol/L) and gelatin(1.50×10^{-2} g/L)
